# Improving the quality of hospital care for children by supportive supervision: a cluster randomized trial, Kyrgyzstan

**DOI:** 10.2471/BLT.16.176982

**Published:** 2016-12-20

**Authors:** Marzia Lazzerini, Venera Shukurova, Marina Davletbaeva, Kubanychbek Monolbaev, Tatiana Kulichenko, Yuri Akoev, Maya Bakradze, Tea Margieva, Ilya Mityushino, Leyla Namazova-Baranova, Elnura Boronbayeva, Aigul Kuttumuratova, Martin Willy Weber, Giorgio Tamburlini

**Affiliations:** a World Health Organization Collaborating Centre for Maternal and Child Health, Institute for Maternal and Child Health IRCCS Burlo Garofolo, Via dell’Istria 65/1, Trieste, 34137, Italy.; b State Medical Institute of Postgraduate and Continuous Training, Bishkek, Kyrgyzstan.; c Republican Clinical Infectious Diseases Hospital, Bishkek, Kyrgyzstan.; d World Health Organization Country Office, Bishkek, Kyrgyzstan.; e Scientific Centre of Children's Health, Moscow, Russian Federation.; f Ministry of Health, Bishkek, Kyrgyzstan.; g Department of Child and Adolescent Health, World Health Organization Regional Office for Europe, Copenhagen, Denmark.

## Abstract

**Objective:**

To determine whether periodic supportive supervision after a training course improved the quality of paediatric hospital care in Kyrgyzstan, where inappropriate care was common but in-hospital postnatal mortality was low.

**Methods:**

In a cluster, randomized, parallel-group trial, 10 public hospitals were allocated to a 4-day World Health Organization (WHO) course on hospital care for children followed by periodic supportive supervision by paediatricians for 1 year, while 10 hospitals had no intervention. We assessed prospectively 10 key indicators of inappropriate paediatric case management, as indicated by WHO guidelines. The primary indicator was the combination of the three indicators: unnecessary hospitalization, increased iatrogenic risk and unnecessary painful procedures. An independent team evaluated the overall quality of care.

**Findings:**

We prospectively reviewed the medical records of 4626 hospitalized children aged 2 to 60 months. In the intervention hospitals, the mean proportion of the primary indicator decreased from 46.9% (95% confidence interval, CI: 24.2 to 68.9) at baseline to 6.8% (95% CI: 1.1 to 12.1) at 1 year, but was unchanged in the control group (45.5%, 95% CI: 25.2 to 67.9, to 64.7%, 95% CI: 43.3 to 86.1). At 1 year, the risk ratio for the primary indicator in the intervention versus the control group was 0.09 (95% CI: 0.06 to 0.13). The proportions of the other nine indicators also decreased in the intervention group (*P* < 0.0001 for all). Overall quality of care improved significantly in intervention hospitals.

**Conclusion:**

Periodic supportive supervision for 1 year after a training course improved both adherence to WHO guidelines on hospital care for children and the overall quality of paediatric care.

## Résumé

**Objectif:**

Déterminer si un encadrement périodique adapté suite à un cours de formation a permis d'améliorer la qualité des soins hospitaliers pédiatriques au Kirghizistan, où il était courant que les soins soient inappropriés, mais où la mortalité postnatale en milieu hospitalier était faible.

**Méthodes:**

Dans le cadre d'un essai en groupes parallèles randomisé par grappes, 10 hôpitaux publics ont bénéficié d'une formation de 4 jours sur les soins hospitaliers pédiatriques dispensée par l'Organisation mondiale de la Santé (OMS), suivie d'un encadrement périodique adapté par des pédiatres pendant 1 an, alors que 10 autres hôpitaux n'ont reçu aucune intervention. Nous avons préalablement évalué 10 indicateurs clés de prise en charge inappropriée de cas pédiatriques, conformément aux directives de l'OMS. L'indicateur principal était la combinaison des trois indicateurs: hospitalisation inutile, risque iatrogène accru et procédures douloureuses inutiles. Une équipe indépendante a évalué la qualité générale des soins.

**Résultats:**

Nous avons préalablement examiné les dossiers médicaux de 4626 enfants hospitalisés âgés de 2 à 60 mois. Dans les hôpitaux ayant bénéficié d'une intervention, la proportion moyenne de l'indicateur principal a chuté de 46,9% (intervalle de confiance, IC, à 95%: de 24,2 à 68,9) à 6,8% (IC à 95%: de 1,1 à 12,1) au bout d'une année, mais est restée inchangée dans le groupe de contrôle. Au bout d'un an, le risque relatif pour l'indicateur principal dans le groupe expérimental par rapport au groupe de contrôle était de 0,09 (IC à 95%: de 0,06 à 0,13). Les proportions des neuf autres indicateurs ont également diminué dans le groupe expérimental (P < 0,0001 pour tous). La qualité générale des soins s'est considérablement améliorée dans les hôpitaux ayant bénéficié d'une intervention.

**Conclusion:**

La mise en place d'un encadrement périodique adapté pendant 1 an à la suite d'un cours de formation permet d'améliorer à la fois la conformité aux directives de l'OMS sur les soins hospitaliers pédiatriques et la qualité générale des soins pédiatriques.

## Resumen

**Objetivo:**

Determinar si la supervisión de apoyo periódica tras un curso de formación mejoró la calidad de la asistencia hospitalaria pediátrica en Kirguistán, donde era común una asistencia poco apropiada pero la mortalidad postnatal hospitalaria era baja.

**Métodos:**

En un ensayo aleatorizado por conglomerados paralelos, se asignó a 10 hospitales públicos un curso de 4 días de la Organización Mundial de la Salud (OMS) sobre asistencia hospitalaria infantil, seguido de una supervisión de apoyo periódica realizada por pediatras durante 1 año, mientras que 10 hospitales no tuvieron ninguna intervención. Prospectivamente, se evaluaron 10 indicadores clave de tratamiento inapropiado de los casos pediátricos, tal como indicaban las directrices de la OMS. El indicador primario fue la combinación de los tres indicadores: hospitalización innecesaria, aumento del riesgo iatrogénico y procedimientos dolorosos innecesarios. Un equipo independiente evaluó la calidad general de la asistencia.

**Resultados:**

Se revisaron prospectivamente los historiales médicos de 4 626 niños hospitalizados de entre 2 y 60 meses. En los hospitales donde se intervino, el porcentaje medio del indicador primario descendió de un 46,9% (intervalo de confianza, IC, del 95%: 24,2 a 68,9) al inicio a un 6,8% (IC del 95%: 1,1 a 12,1) al cabo de un año, pero no cambió en el grupo de control. Al cabo de un año, el coeficiente de riesgo del indicador primario en el grupo de intervención, en comparación con el grupo de control, fue del 0,09 (IC del 95%: 0,06 a 0,13). Los porcentajes de los otros nueve indicadores también se redujeron en el grupo de intervención (*P* < 0,0001 para todos). La calidad general de la asistencia mejoró de forma significativa en los hospitales donde se intervino.

**Conclusión:**

La supervisión de apoyo periódica durante un año tras un curso de formación mejoró tanto la adherencia a las directrices de la OMS sobre asistencia hospitalaria infantil como la calidad general de la asistencia pediátrica.

## ملخص

الغرض الوقوف على نجاح الإشراف الداعم الدوري المُقدّم بعد إجراء دورة تدريبية في تحسين جودة الرعاية المقدمة للأطفال في المستشفيات بقيرغيزستان في الأماكن التي يشيع فيها غياب توفير الرعاية المناسبة مع تدني نسبة الوفيات بعد الولادة في المستشفيات.

**الطريقة:**

في تجربة عنقودية وعشوائية أجريت على مجموعة موازية، تم تخصيص 10 مستشفيات عمومية لإجراء دورة تدريبية تابعة لمنظمة الصحة العالمية لمدة 4 أيام بشأن الرعاية المقدمة للأطفال في المستشفى في أعقاب توفير الإشراف الدوري الداعم من جانب أطباء الأطفال لمدة عام واحد، بينما لم تخضع أي من المستشفيات العشرة لعمليات التدخل. قمنا بتقييم 10 مؤشرات رئيسية محتملة لإدارة حالات الأطفال بطريقة غير مناسبة، كما هو موضح في المبادئ التوجيهية الخاصة بمنظمة الصحة العالمية. وكان المؤشر الأساسي يتمثل في الجمع ما بين ثلاثة مؤشرات: عدم وجود ضرورة للاحتجاز بالمستشفى، وزيادة نسبة الخطر الناشئة عن الأخطاء في العلاج، والخضوع لإجراءات مؤلمة غير ضرورية. وقام فريق مستقل بتقييم المستوى الإجمالي للجودة النوعية للرعاية المقدمة.

**النتائج:**

قمنا بإجراء مراجعة استباقية للسجلات الطبية للأطفال الذين تم علاجهم بالمستشفى والبالغ عددهم 4626 طفلاً والذين تتراوح أعمارهم من شهرين إلى 60 شهرًا. في المستشفيات الخاضعة للتدخل، انخفض متوسط نسبة المؤشر الأساسي من ‏46.9% (بنسبة أرجحية مقدارها 95‏%: 24.2 إلى 68.9) عند خط الأساس إلى 6.8‏% (بنسبة أرجحية مقدارها 95‏%: 1.1 إلى 12.1) في عام واحد، ولكن لم تتغير النسبة في مجموعة الشاهد. في عام واحد، كان معدل الخطورة للمؤشر الأساسي في التدخل مقابل مجموعة الشاهد يبلغ 0.09 (بنسبة أرجحية مقدارها ‏95%: 0.06 إلى 0.13). انخفضت أيضًا نسب حدوث المؤشرات التسعة الأخرى في مجموعة التدخل (الاحتمال < 0.0001 للجميع). وقد تحسن المستوى الإجمالي للجودة النوعية للرعاية المقدمة بشكلٍ كبير في المستشفيات الخاضعة للتدخل.

**الاستنتاج:**

ساهم توفير الإشراف الداعم الدوري لعام واحد بعد إجراء دورة تدريبية في تحسين خطوات الالتزام بالمبادئ التوجيهية الخاصة بمنظمة الصحة العالمية بشأن الرعاية المقدمة للأطفال في المستشفى والمستوى الإجمالي للجودة النوعية للرعاية المقدمة للأطفال على حدٍ سواء.

## 摘要

目的 旨在确定培训课程结束后开展定期支持性监督是否能够改善吉尔吉斯斯坦儿童医院护理的质量。在吉尔吉斯斯坦,护理不当很常见,但住院产后死亡率较低。

方法 在一项整群随机平行组试验中,安排 10 家公立医院开展为期 4 天的世界卫生组织 (WHO) 儿童医院护理课程,随后由儿科医生进行为期 1 年的定期支持性监督,在此期间,不对 10 家医院进行任何干预。 按照 WHO 指南的说明,我们对不当儿科病历管理的 10 项关键指标进行了预期性评估。 主要指标为以下三项指标的组合: 不必要的住院治疗、医源性风险增加以及不必要的痛苦疗程。 一个独立团队对整体护理质量进行了评估。

结果 我们预先审查了 4626 名 2 个月至 60 个月大住院儿童的医疗记录。 在干预医院,1 年间主要指标的平均比例从基准线下 46.9% (95% 置信区间,CI: 24.2-68.9)下降至 6.8% (95% CI: 1.1-12.1),但对照组无变化。 1 年后,干预组与对照组主要指标的风险比为 0.09 (95% CI: 0.06-0.13)。 干预组中的其它九项指标比例也有所下降(全部 P  < 0.0001)。 干预医院的整体护理质量得到了显著改善。

结论 课程结束后进行为期 1 年的定期支持性监督既增强了对世界卫生组织 (WHO) 儿童医院护理方面指南的遵守程度,同时也改善了儿科护理的整体质量。

## Резюме

**Цель:**

Определить, способствует ли периодический дополнительный надзор после проведения обучающего курса повышению качества оказания стационарной педиатрической помощи в Кыргызстане, где среднестатистический уровень качества оказания медицинской помощи был невысоким, но уровень внутристационарной постнатальной смертности был низким.

**Методы:**

В ходе кластерного рандомизированного исследования с использованием параллельных групп было определено 10 государственных больниц для проведения 4-дневного обучающего курса Всемирной организации здравоохранения (ВОЗ) по оказанию стационарной медицинской помощи детям с проведением последующего периодического дополнительного надзора педиатрами в течение 1 года, в то время как в 10 других больницах не было проведено никакого вмешательства. Мы провели перспективную оценку 10 ключевых показателей ненадлежащего качества ведения педиатрических пациентов в соответствии с указаниями руководящих принципов ВОЗ. Основным показателем являлось сочетание трех факторов: госпитализации без необходимости, повышенного ятрогенного риска и проведения ненужных болезненных процедур. Общее качество оказания медицинской помощи оценивалось независимой группой.

**Результаты:**

Мы в перспективном порядке проверили медицинские отчеты о 4626 госпитализированных детях в возрасте от 2 до 60 месяцев. В больницах с вмешательством средняя доля основного показателя за 1 год снизилась с 46,9% (95%-й доверительный интервал, ДИ: от 24,2 до 68,9) на исходном уровне до 6,8% (95%-й ДИ: от 1,1 до 12,1), но осталась неизменной в контрольной группе. В течение 1 года относительный риск для основного показателя в группе с вмешательством по сравнению с контрольной группой составил 0,09 (95%-й ДИ: от 0,06 до 0,13). Доли остальных девяти показателей также снизились в группе с вмешательством (*P* < 0,0001 для всех). В целом качество медицинской помощи значительно улучшилось в больницах с вмешательством.

**Вывод:**

Периодический дополнительный надзор в течение 1 года после обучающего курса помог улучшить как соблюдение требований руководящих принципов ВОЗ по стационарному медицинскому уходу за детьми, так и общее качество оказания медицинской помощи детям.

## Introduction

Hospital care of an adequate quality is essential for health and well-being and is a basic component of human rights.^1^^,^^2^ Moreover, reducing inequalities in the quality of care is a primary objective of *Health 2020*,^1^ a strategic policy document issued by the 53 Member States in the World Health Organization’s (WHO) European Region.^1^ Although data on the quality of hospital care for children in countries with constrained resources are starting to accumulate,^3^^–^^16^ very little is known about which interventions are most effective for enhancing quality. The approach most commonly advocated by WHO for improving case management for common children’s diseases has been the dissemination of evidence-based guidelines, usually combined with staff training.^4^^–^^7^^,^^17^ Many countries implementing the Integrated Management of Childhood Illness strategy developed by WHO and the United Nations Children’s Fund have adopted this approach. However, recent evidence indicates that training alone does not ensure better case management and, even after training, it may be difficult to incorporate new knowledge into practice.^18^^–^^20^ Supportive supervision for staff has been proposed as an intervention for increasing adherence to clinical guidelines.^20^^–^^22^ However, little high-quality evidence, for example from randomized controlled trials, is available on the efficacy of supportive supervision in general or, more specifically, on its use for improving the quality of hospital care for common childhood conditions in low- and middle-income countries.^17^

Previous assessments of the quality of paediatric care in the Commonwealth of Independent States showed that in-hospital postnatal case fatality rates were low but inappropriate case management was common and characterized by unnecessary hospitalization, overdiagnosis and overtreatment, possibly associated with wasted resources and a risk to children’s health.^3^^–^^6^ The main underlying reasons were a lack of up-to-date, evidence-based guidelines and the persistence of outdated clinical practices.^3^^–^^6^

In Kyrgyzstan, a lower-middle-income country in the WHO European Region, the mortality rate in children younger than 5 years has decreased steadily from 63 per 1000 live births in 1994 to 24 per 1000 in 2013.^23^ In 2012, the Kyrgyz Ministry of Health and WHO agreed to implement a project aimed at improving the quality of hospital paediatric care. This project included an impact evaluation in the form of a cluster randomized controlled trial.

This paper presents the findings of this cluster randomized controlled trial, which was designed to determine whether periodic supportive supervision, provided after a standard WHO 4-day training course, improves paediatric case management in hospitals and increases the overall quality of care for common childhood conditions.

## Methods

**Fig. 1:**
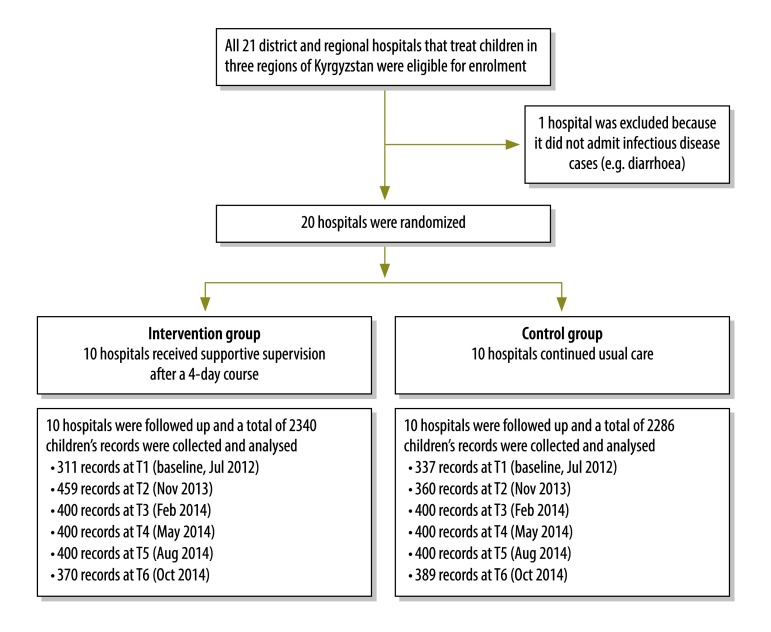
Flow diagram, study on improving paediatric hospital care by supportive supervision of staff, Kyrgyzstan, 2012–2014

**Fig. 2:**
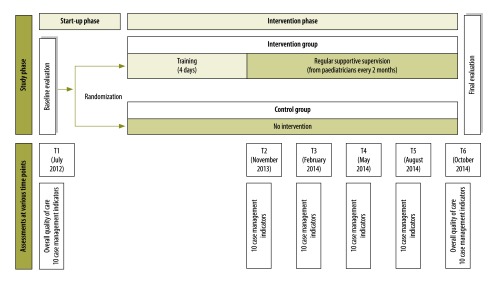
Schedule, study on improving paediatric hospital care by supportive supervision of staff, Kyrgyzstan, 2012–2014

Notes: The overall quality of care was assessed using the World Health Organization’s *Hospital care for children: quality assessment and improvement tool*^24^ and the *Pocket book of hospital care for children: guidelines for the management of common illnesses with limited resources*.^25^ The 10 case management indicators were also obtained from *Hospital care for children: quality assessment and improvement tool*.^24^ The assessment at time point T2 took place shortly after the training course and at almost the same time as the first supportive supervision visit.

We conducted a cluster, randomized, parallel-group trial in the Chui, Issyk-Kul and Talas Regions of northern Kyrgyzstan and involved 20 district and regional public hospitals that treated children (Fig. 1). Hospitals were taken as the unit of randomization to avoid contamination between practitioners at the same location. After a baseline assessment using WHO’s *Hospital care for children: quality assessment and improvement tool*,^24^ which compares case management with guidelines in WHO’s *Pocket book of hospital care for children*,^25^ 10 hospitals were randomized to the intervention while 10 continued with usual care (Fig. 2). For randomization, hospitals were first stratified by geographical distribution (i.e. west versus east) and by type (i.e. regional versus district hospitals), then randomized by extraction of opaque sealed envelopes prepared by WHO.

T: time point.

Note: The intervention comprised periodic supportive supervision for 1 year after a standard World Health Organization 4-day training course.

Doctors in charge of children from the intervention hospitals attended a 4-day, WHO training course on WHO’s guidelines on hospital care for children.^25^ In collaboration with the health ministry, the WHO country office coordinated the course, which was held in the capital Bishkek, supervised by the WHO Regional Office for Europe. Subsequently, a team of eight senior national paediatricians, who had undergone training on both WHO’s guidelines and supportive supervision methods, provided supervision for doctors, nurses and managers every 2 months for 1 year. The WHO country office regularly checked that supportive supervision was provided on time. During each hospital visit, two paediatricians provided supportive supervision over 1 or 2 days based on a peer-to peer, plan–do–study–act model,^26^ which involved: (i) identifying and agreeing on the actions needed to improve the quality of care; (ii) implementing those actions; (iii) monitoring progress; and (iv) discussing any additional actions needed. Document templates were used for these activities and training was reinforced at each subsequent visit.

### Outcomes

The primary aim of the study was to determine whether or not case management was inappropriate when compared with guidelines in WHO’s *Pocket book of hospital care for children*;^25^ this was evaluated using 10 key indicators in WHO’s *Hospital care for children: quality assessment and improvement tool* (Table 1).^24^ Our primary indicator was the combination of the three indicators: unnecessary hospitalization, an increased risk of iatrogenic effects and unnecessary painful procedures. Our primary study outcome was the change in the mean proportion of hospitalized children, who had medical records documenting the primary indicator. For both intervention and control groups, 10 trained data collectors obtained data prospectively from paper-based medical records on the 10 indicators. Data collection time points (T) were designated T1 (July 2012), T2 (November 2013), T3 (February 2014), T4 (May 2014), T5 (August 2014) and T6 (October 2014; Fig. 2). At each time point, data collectors examined in each hospital the medical records of a random sample of 35 to 40 children aged 2 to 60 months who had been hospitalized in the previous 2 months at each hospital and who presented with: (i) a cough or breathing difficulties; (ii) diarrhoea; or (iii) fever – all common in childhood. Data collectors filled in a paper-based template for the indicators. Subsequently, two data collector coordinators transferred this information into a predefined electronic spreadsheet, which they then sent to the study investigators by email. The study investigators checked the spreadsheets for internal consistency after each data collection.

**Table 1:** Indicators of inappropriate paediatric case management, WHO ***Quality assessment and improvement tool for hospital care for children***^24^

Indicator	Definition	Examples
Combined negative indicator (primary indicator)	Concomitant presence in the same child of: (i) unnecessary hospitalization; (ii) increased iatrogenic risk; and (iii) unnecessary painful procedures	See descriptions of individual components
Unnecessary hospitalization	Failure to comply with recommendations on hospitalization in WHO’s *Pocket book of hospital care for children*^25^	(i) The child had “no pneumonia: cough or cold” according to WHO pocket book criteria and did not satisfy the criteria for “severe pneumonia” (thereby requiring hospitalization) but was hospitalized; (ii) the child had “some dehydration” according to WHO criteria and did not satisfy the criteria for “severe dehydration” but was hospitalized
Incorrect diagnosis	Failure to comply with WHO pocket book recommendations on diagnosis	(i) The child had “no pneumonia: cough or cold” according to WHO pocket book criteria but was diagnosed with “pneumonia” or “severe pneumonia”; (ii) the child had “some dehydration” according to WHO criteria but was diagnosed with “severe dehydration”
Incorrect treatment	Failure to comply with WHO pocket book recommendations on treatment	(i) The child should have received treatment for “no pneumonia: cough or cold” according to WHO pocket book criteria but was treated for “pneumonia” or “severe pneumonia”; (ii) the child had “some dehydration“ according to WHO criteria but was treated for “severe dehydration”
Inconsistent diagnosis and treatment	There was no consistency between diagnosis and treatment or the diagnosis was not described clearly in the patient’s chart	(i) The child was diagnosed with “pneumonia” according to WHO pocket book criteria but was treated for “severe pneumonia”; (ii) the child had “some dehydration“ according to WHO criteria but was treated for “severe dehydration”
Increased iatrogenic risk	Administration of two or more unnecessary drugs as indicated by a failure to comply with WHO pocket book recommendations on case management	The administration of any unnecessary drug (i.e. a drug not recommended by the WHO pocket book) whose efficacy was not proven and which had possible adverse effects, e.g. steroids or antibiotics for diarrhoea, sedatives for children with fever and so-called cardiotonic or neuroprotective drugs in children without a clear indication for such treatment
Unnecessary painful procedures	Performance of unnecessary invasive procedures as indicated by a failure to comply with WHO pocket book recommendations on case management	(i) Intramuscular or intravenous antibiotic injections in a child who, according to WHO pocket book criteria, should have been treated with an oral antibiotic (e.g. a child with “pneumonia” and no vomiting or signs of “severe pneumonia”); (ii) administration of intravenous fluids to a child who, according to WHO criteria, could have been rehydrated orally (e.g. a child with diarrhoea but with “no dehydration” or “some dehydration” according to WHO criteria and without repetitive vomiting
Inadequate monitoring	Inadequate monitoring for the clinical diagnosis received, as indicated by WHO pocket book criteria	(i) Failure to monitor the respiratory rate at least twice a day in a child with a respiratory infection; (ii) failure to monitor weight at least twice a day in a child with diarrhoea; (iii) failure to monitor neurological status at least twice a day in a child with meningitis
Nutritional status not assessed	Failure to assess growth or identify malnutrition	(i) Failure to assess a child’s growth adequately, as indicated by WHO pocket book criteria (i.e. measurement of both weight-for-age and height-for-age and comparison with WHO 2006 growth standards); (ii) failure to identify a child with acute or chronic malnutrition according to WHO criteria
Incorrect use of intravenous fluids	Fluids not used in accordance with WHO pocket book recommendations	(i) Intravenous fluids were prescribed when they were not needed (e.g. when the child was able to drink); (ii) the wrong type of fluid was given (e.g. a hypotonic solution); (iii) the wrong quantity was given (i.e. too much or too little); (iv) fluids were given at the wrong interval

WHO: World Health Organization.

At the baseline assessment (i.e. time point T1), health staff, data collectors, patients and data analysts were all blinded to the study allocation group. During the intervention (i.e. T2 to T6), neither hospital staff nor the supportive supervision team could be blinded but they were not involved in data collection or data analysis. Data collectors knew the allocation group but were not involved in data analysis. However, children and their families were blinded to both the allocation group and the characteristics of the intervention.

In addition, an independent team of international consultant paediatricians assessed the overall quality of care delivered at each hospital at time points T1 and T6 using WHO’s *Hospital care for children: quality assessment and improvement tool* (Fig. 2), adapted for use in the WHO European Region.^24^ The tool systematically assesses different components of the health system that contribute to quality of care – it includes three main sections and a total of 17 subsections. The main sections are: (i) hospital support systems; (ii) case management; and (iii) policies and organization of care. Using structured checklists in the assessment tool, the team attributed a score to each of approximately 250 items based on standards derived from WHO recommendations and other relevant guidelines.^24^^,^^25^ During the evaluations, a summary score was derived for each of the 17 subsections: it ranged from 0 for totally inadequate care to 3 for care that met international standards.

To ensure data quality, we (i) field-tested the template used for collecting data on indicators of inappropriate case management, which included an explicit definition of each indicator, before being used in the study; (ii) field-tested data collectors’ performance before the study and their understanding was reinforced, if necessary, to ensure consistency; (iii) rotated data collectors among hospitals to reduce the influence of subjectivity; (iv) determined the reliability of the data collectors in evaluating medical records by comparing their evaluations with those of a team of independent international paediatricians at time points T1, T2 and T6 (Fig. 2); and (v) monitored the completeness and internal consistency of the data collected by an external independent data analyst at regular intervals after each supportive supervision visit.

The ethical committees of the Kyrgyz State Medical Institute of Postgraduate and Continuous Training and of the Institute for Maternal and Child Health IRCCS Burlo Garofolo in Italy approved the study. In addition, the director of each participating hospital gave informed consent. The privacy of children and their families was protected by collecting data anonymously. Data are reported in accordance with the Consolidated Standards of Reporting Trials (CONSORT) statement for cluster randomized controlled trial^27^ and the trial is registered with ClinicalTrials.gov under the identifier NCT02001116.

### Statistical analysis

Using data from the baseline assessment study, we calculated the sample size required by taking into account: (i) the fixed number of clusters; (ii) the intracluster correlation coefficient determined at the baseline assessment; (iii) the expected effect of the intervention; and (iv) the desired power of the study.^28^^,^^29^ In the baseline assessment, the proportion of the primary indicator was 45% and the intracluster correlation coefficient was 0.16. Under the hypothesis that the proportion of children who satisfied the conditions for the primary indicator would decrease after the intervention by 35 percentage points in the intervention group and by 10 percentage points in the control group, we estimated that a total of 640 children (i.e. 32 at each of the 20 facilities) had to be evaluated at each time point to detect a significant difference between the two groups with a power of 80% and a significance of 90% (α = 5%, two-sided test).

We present categorical variables as absolute numbers, proportions and risk ratios (RRs) with 95% confidence intervals (CIs) and quantitative variables as means and standard deviations. We used Fisher’s exact test or Yates’s corrected *χ*^2^ test, as appropriate, to compare categorical variables and *t* test and the mean difference to compare quantitative variables. Trends in proportions were compared using the Cochran–Armitage *χ*^2^ test. All statistical tests were two-sided and a *P*-value less than 0.05 was considered statistically significant. We analysed data using Stata version 12 (StataCorp. LP, College Station, United States of America) and OpenEpi version 2.3.1 (Andrew G Dean and Kevin M Sullivan, Atlanta, USA).

## Results

Table 2 gives details of the hospitals involved in the study. There was no significant difference in any characteristic between intervention and control groups. In total, data on 4626 children were collected and analysed over the six time points: 2340 in the intervention group and 2286 in the control group. At baseline, there was no significant difference between the groups for any of the 10 indicators of inappropriate case management.

**Table 2:** Hospital characteristics, study on improving paediatric hospital care by supportive supervision of staff, Kyrgyzstan, 2012–2014

Characteristic at study baseline	Intervention group													Control group											
		Hospital 1	Hospital 2	Hospital 3	Hospital 4	Hospital 5	Hospital 6	Hospital 7	Hospital 8	Hospital 9	Hospital 10	Mean (SD)		Hospital 1	Hospital 2	Hospital 3	Hospital 4	Hospital 5	Hospital 6	Hospital 7	Hospital 8	Hospital 9	Hospital 10	Mean (SD)	
Catchment population, no. × 1000		180	230	57	46	159	85	79	42	50	136	106 (66)		183	94	42	58	29	76	63	59	39	56		70 (44)
Catchment population younger than 18 years of age, no. × 1000		96	89	19	15	48	27	31	13	14	38	39 (31)		46	25	11	22	12	26	25	22	10	22		22 (10)
Child beds, no.		53	90	16	20	28	23	17	15	25	29	32 (23)		30	61	20	10	10	15	30	20	22	22		24 (15)
Paediatricians, no.		4	9	1	1	4	3	2	1	3	3	3 (2)		3	9	2	1	1	3	3	3	2	3		3 (2)
Other children’s doctors, no.		4	19	15	36	10	9	17	12	3	25	15 (10)		7	18	14	5	10	26	16	14	18	37		17 (9)
All children’s doctors, no.		8	28	16	37	14	12	19	13	6	28	18 (10)		10	27	16	6	11	29	19	17	20	40		20 (10)
Children’s nurses, no.		43	80	24	6	24	26	22	105	72	66	47 (32)		15	46	20	12	15	70	25	30	37	170		44 (48)
Hospital visits by children younger than 18 years, no. per year		4477	8033	864	1026	1854	1607	1005	1030	802	1685	2238 (2 305)		2034	4439	2220	900	549	940	1230	1068	665	3395		1744 (1 289)
Hospital visits by children younger than 5 years, no. per year		1752	2769	684	162	793	1418	843	402	389	1316	1052 (784)		452	2020	1405	42	192	219	581	435	291	1320		695 (654)
Admissions of children younger than 18 years, no. per year		3067	5199	480	1026	1363	1607	1104	736	663	1596	1684 (1 435)		1247	4062	734	718	468	870	1168	873	613	2837		1359 (1 162)
Admissions of children younger than 5 years, no. per year		908	1664	360	162	531	1280	387	327	362	1266	724 (517)		1256	1785	341	186	184	178	558	367	273	1046		617 (556)

SD: standard deviation.

There was no significant difference between intervention and control group means for any variable.

**Fig. 3:**
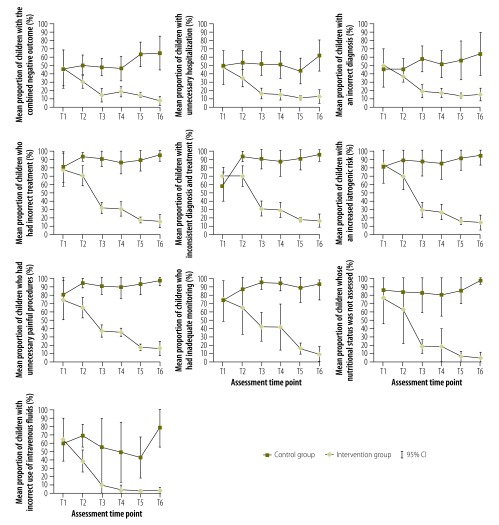
Proportion of children with one of the 10 indicators of inappropriate paediatric case management, study on improving paediatric hospital care by supportive supervision of staff, Kyrgyzstan, 2012–2014

During the study period, similar trends were observed for all indicators (Fig. 3). In particular, the primary indicator decreased significantly from 46.9% (95% CI: 24.2 to 68.9) at T1 to 6.8% (95% CI: 1.1 to 12.1) at T6 (*P* for trend < 0.0001) in the intervention hospitals. No significant change was observed in control hospitals, 45.5% (95% CI: 25.2 to 67.9) at T1 to 64.7% (95% CI: 43.3 to 86.1) at T6 (*P* for trend > 0.05). At T6, the RR for the primary indicator in the intervention versus the control group was 0.09 (95% CI: 0.06 to 0.13). The mean proportion of unnecessary hospitalization also decreased over time in the intervention group from 47.6% (95% CI: 27.5 to 67.7) at T1 to 13.1% (95% CI: 5.0 to 21.2) at T6 (*P* for trend < 0.001), but remained stable in the control group (RR in the intervention versus the control group at T6: 0.23; 95% CI: 0.17 to 0.30). Similarly, the mean proportion of an incorrect diagnosis decreased significantly in the intervention group from 49.7% (95% CI: 29.9 to 69.5) at T1 to 14.7% (95% CI: 7.0 to 22.4) at T6 (*P* for trend < 0.0001), but not in the control group (RR in the intervention group at T6: 0.22; 95% CI: 0.17 to 0.29). The mean proportion of incorrect treatment decreased significantly only in the intervention group, from 77.9% (95% CI: 57.6 to 98.2) at T1 to 15.7% (95% CI: 7.4 to 24.0) at T6 (*P* for trend < 0.0001); the RR in the intervention group at T6 was 0.16 (95% CI: 0.12 to 0.29). A similar pattern was observed for the other six indicators and the RR in the intervention versus the control group was: 0.16 (95% CI: 0.13 to 0.21) for an inconsistency between diagnosis and treatment; 0.14 (95% CI: 0.11 to 0.19) for increased iatrogenic risk; 0.15 (95% CI: 0.12 to 0.20) for unnecessary painful procedures; 0.09 (95% CI: 0.07 to 0.13) for inadequate monitoring; 0.05 (95% CI: 0.03 to 0.08) for failure to assess nutritional status; and 0.03 (95% CI: 0.02 to 0.06) for inadequate use of fluids.

CI: confidence interval.

Notes: Groups were randomized and each group consisted of 10 hospitals. The timing of assessments at time points T1 to T6 is described in Fig. 2. The intervention comprised periodic supportive supervision for 1 year after a standard World Health Organization 4-day training course. Details of the number of children’s medical records examined at each time point are given in Fig. 1. The combined negative indicator was the combination of the three indicators of: (i) unnecessary hospitalization; (ii) increased iatrogenic risk; and (iii) unnecessary painful procedures. The indicators were obtained from the World Health Organization’s *Hospital care for children: quality assessment and improvement tool*.^24^ and are described in Table 1.

At baseline, there was no significant difference between intervention and control hospitals in quality-of-care summary scores for the 17 subsections of WHO’s *Hospital care for children: quality assessment and improvement tool*.^24^ In contrast, summary scores at the end of the intervention period were significantly higher in the intervention than the control group (Table 3). Additional details of the changes observed and of residual problems that are amenable to future quality improvements are available from the corresponding author.

**Table 3:** Quality of children’s care, by study group, study on improving paediatric hospital care by supportive supervision of staff, Kyrgyzstan, 2012–2014

Quality-of-care category^a^	Summary score					
	Baseline			End of the study		
	Intervention group, mean (SD)	Control group, mean (SD)	Intervention versus control group, mean difference (95% CI)	Intervention group, mean (SD)	Control group, mean (SD)	Intervention versus control group, mean difference (95% CI)
**Hospital support systems**						
Information systems and statistics	1.85 (0.44)	1.70 (0.53)	0.15 (−0.30 to 0.60)	2.68 (0.24)	1.55 (0.52)	1.26 (0.85 to 1.66)
Drugs and equipment	2.27 (0.61)	2.00 (0.73)	0.27 (−0.36 to 0.90)	2.94 (0.11)	1.68 (0.60)	1.13 (0.74 to 1.51)
Laboratory	2.50 (0.46)	2.33 (0.57)	0.17 (−0.31 to 0.66)	2.93 (0.12)	2.28 (0.80)	0.65 (0.11 to 1.18)
Emergency support	1.69 (0.28)	1.51 (0.33)	0.18 (−0.10 to 0.46)	2.85 (0.13)	1.27 (0.50)	1.58 (0.23 to 1.92)
Paediatric ward	1.65 (0.40)	1.54 (0.55)	0.11 (−0.34 to 0.56)	2.85 (0.19)	1.60 (0.50)	1.25 (0.89 to 1.60)
**Case management**						
Cough and breathing difficulties	1.02 (0.63)	0.99 (0.51)	0.01 (−0.52 to −0.54)	2.53 (0.33)	1.17 (0.62)	1.36 (0.89 to 1.82)
Diarrhoea	0.65 (0.58)	0.60 (0.54)	0.05 (−0.47 to 0.57)	2.70 (0.21)	1.12 (0.48)	1.58 (0.23 to 1.92)
Anaemia	1.12 (0.37)	1.09 (0.43)	0.03 (−0.25 to 0.31)	2.28 (0.31)	0.98 (0.70)	1.30 (0.79 to 1.80)
Febrile conditions	0.87 (0.43)	0.96 (0.39)	−0.09 (−0.47 to 0.29)	2.12 (0.31)	1.16 (0.37)	0.96 (0.63 to 1.28)
Chronic conditions	1.72 (0.67)	2.17 (0.29)	−0.45 (−0.93 to 0.03)	1.73 (0.35)	0.97 (0.31)	1.76 (0.44 to 1.07)
Surgery	1.46 (0.39)	1.29 (0.26)	0.17 (−0.14 to 0.48)	2.88 (0.17)	1.40 (0.59)	1.48 (1.07 to 1.88)
**Policies and organization of care**						
Supportive care	1.00 (0.56)	1.08 (0.48)	−0.08 (−0.57 to 0.41)	2.87 (0.13)	1.25 (0.55)	1.62 (0.24 to 1.99)
Child-friendly services	1.25 (0.32)	1.43 (0.36)	−0.18 (−0.50 to 0.14)	2.69 (0.26)	1.20 (0.44)	1.49 (1.15 to 1.82)
Monitoring	0.96 (0.78)	1.20 (0.57)	−0.24 (−0.88 to 0.40)	2.76 (0.21)	0.91 (0.57)	1.85 (1.84 to 2.25)
Auditing and guidelines	1.74 (0.51)	1.69 (0.31)	0.05 (−0.34 to 0.44)	2.73 (0.19)	1.56 (0.74)	1.17 (0.66 to 1.67)
Access to hospital	2.00 (0.28)	2.03 (0.43)	−0.03 (−0.37 to 0.31)	2.76 (0.26)	1.85 (0.37)	0.91 (0.60 to 1.21)
Mothers’ satisfaction	1.72 (0.62)	1.70 (0.32)	0.02 (−0.44 to 0.48)	2.83 (0.19)	1.89 (0.23)	0.94 (0.74 to 1.13)

CI: confidence interval; SD: standard deviation.

^a^ Quality-of-care categories relate to sections and subsections of the World Health Organization’s *Hospital care for children: quality assessment and improvement tool*.^24^

## Discussion

We found that a standard, 4-day, WHO training course followed by periodic supportive supervision from national, trained paediatricians every 2 months for 1 year significantly reduced inappropriate case management of hospitalized children and improved the overall quality of paediatric care. Our findings add to the existing literature^30^^–^^32^ and indicate that supportive supervision can improve both adherence to clinical guidelines and the overall quality of care.

Our study was a pragmatic trial because it was conceived as part of an implementation project rather than being performed in a so-called pure study setting. Nevertheless, the supportive supervision team comprised highly motivated and trained staff and external monitoring was carried out regularly to ensure that supportive supervision was provided on time. The results of the study cannot be directly generalized to situations in which a lack of equipment, drugs, supplies or human resources is a major problem or to places where a different form of supportive supervision is being used (e.g. lower-intensity supportive supervision, less-well-trained staff or no external monitoring).

In line with other studies on quality improvement interventions, our outcomes were process outcomes (i.e. indicators of case management quality compared with reference guidelines) rather than health outcomes.^30^^,^^31^ We did not assess mortality or morbidity because in-hospital, postneonatal mortality in countries in the WHO European Region is too low to be used for evaluating interventions and because, to be reliable, data on morbidity (such as the proportion of children with complications from common diseases) should be collected at hospital, primary care and community levels. For this study, we selected the measures of inappropriate case management on the basis of previous experience with evaluating the quality of hospital care in countries in the Commonwealth of Independent States, which showed that unnecessary hospitalization, incorrect diagnosis, incorrect treatment, a lack of monitoring and a lack of attention to comprehensive care (e.g. to nutrition) were the main problems.^3^^–^^6^ These assessment methods are the most reliable and universally accepted, standardized, quantitative indicators currently available for evaluating the quality of paediatric hospital care in settings where mortality is low. Should better indicators become available in the future, they should be considered for use in further research.

A limitation of the study is that the data collectors were not blinded to the allocation group. However, information about the 10 indicators of inappropriate case management was collected from medical records, which are legal documents and, as such, should be considered reliable, and the assessment was based on predefined case definitions, criteria and reference standards,^25^ In addition, the reliability of the data collectors’ evaluations of medical records was examined at time points T1, T2 and T6 and found to be very high: Cohen’s kappa coefficient for inter-rater agreement between data collectors and a team of international independent paediatricians was 0.82, 0.89 and 0.91 at the three time points, respectively. Moreover, it is unlikely that the large differences in quality of care observed between intervention and control hospitals can be explained by the influence of subjectivity.

We did not include cost, patient satisfaction or the satisfaction of health-care providers as study outcomes. However, the administrative data collected suggested that the cost of drugs, especially parenteral drugs, in most intervention hospitals had decreased and interviews with staff and patients indicated anecdotally that satisfaction had increased. Future studies should include a cost–effectiveness analysis and evaluate staff and patient satisfaction. In addition, future research should investigate which approach to supportive supervision results in the greatest improvements in different settings. The existing literature suggests that supportive supervision must be tailored to the local context to some extent. For example, low- or moderate-intensity supportive supervision can be effective in some settings, whereas high-intensity supervision may be needed in others to bring about real changes in behaviour, knowledge and practices.^19^^–^^21^^,^^30^^–^^33^ In our study, improvements in all indicators of inappropriate case management were already apparent by time point T3 (i.e. within 6 months of the first supportive supervision visit), which suggested that the intensity of supportive supervision could be decreased in the following period should improvements be observed.

The ideal providers of supportive supervision may vary according to the setting. In our study and a study in Kenya,^30^ the supportive supervision team comprised paediatricians; of two trials performed in primary care, the providers were paediatricians in one^31^ and medical officers and staff supervising the Integrated Management of Childhood Illness in the other.^32^ The characteristics of the ideal provider may depend on who is receiving supportive supervision: adequately trained medical officers may be able to provide effective supportive supervision to primary care staff, whereas a team of experienced paediatricians may be needed for hospital staff.

The intervention hospitals in our study should be further assessed after a longer period of time to determine whether the improvements observed are maintained. Future studies in these hospitals and in different contexts elsewhere should identify the most cost-effective way of providing effective supportive supervision over the long term. Furthermore, projects should also aim to improve case management in primary care and to strengthen referral systems, thereby ensuring better coordination between different levels of care. Our results indicate that policy-makers should consider using supportive supervision to increase adherence to evidence-based guidelines for paediatric hospital care.
